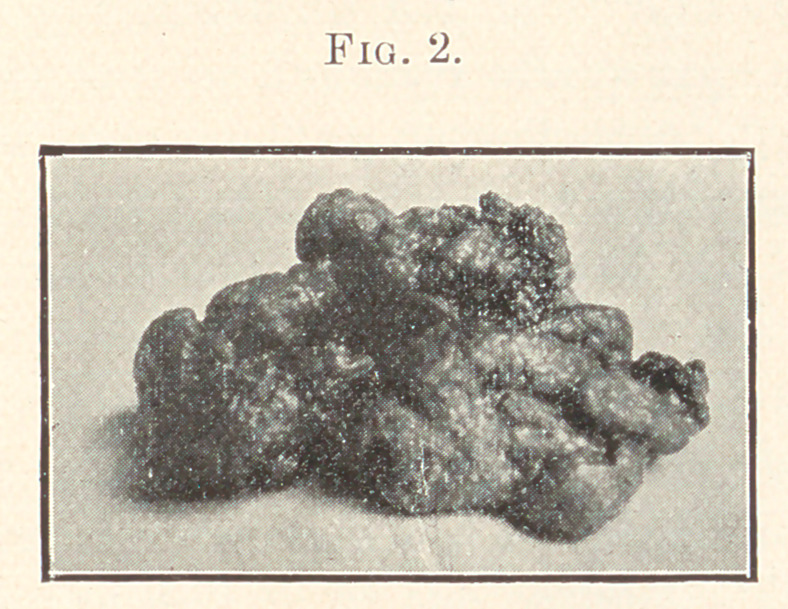# An Interesting Case of Exostosis; Asepsis in Operations

**Published:** 1894-08

**Authors:** Albert Westlake

**Affiliations:** New York


### AN INTERESTING CASE OF EXOSTOSIS; ASEPSIS IN OPERATIONS.

BY ALBERT WESTLAKE, D.D.S., NEW YORK.

Tumors of the oral cavity are always an interesting study to the dental as well as to the general surgeon.

Those of our own profession, whose environments are in large cities and are associated in hospital work, frequently have specially abnormal conditions of this region come within their observation and care.

I do not consider that our dental colleges give any too much time and attention in their curriculum to the physiological and pathological conditions of the jaw and its surrounding tissue.

The diseases of the mouth is a study of vast possibilities, and should be dealt with in an equal, if not more exhaustive, manner than the mere mechanical filling of teeth, as such teaching will lead to an improved aseptic treatment of these parts by the graduate. Numerous cases have been cited in the past, and the librarians of our hospitals can greatly enlarge the list, where the malignant growths in the mouth can be traced to failure of cleanliness in dental operations.

This may be an "old saw," but the changes should be rung out on this subject to not only impress our brethren in the smaller cities and towns, but many of our metropolitan practitioners with the importance of thorough asepsis in both instruments and materials. When we witness the minute attention given such details by our general surgeons in even minor operations, we feel a sense of regret that the majority of the matriculates of our dental colleges do not avail themselves of its refreshing influence by attending the public operations in the amphitheatres of our hospitals. On the theory that cleanliness is next to godliness, the millennium is approaching through the surgical profession.

In my association with the surgeons in private operations, I have found the same adherence to absolute cleanliness that they exhibit in hospital work.

I have presented these thoughts as a prelude to reporting an interesting case as represented by the accompanying figure. Although of a benign nature, the removal of the tumor occupied probably not more than fifteen minutes, yet the details connected with its excision were as complete as was exhibited in the extirpation of a tongue by the same surgeon which I witnessed at the post-graduate hospital one hour later.

The various instruments were previously boiled in soda solutions for one hour and a half, and brought to the private house where the operation was to be performed in a well-arranged receptacle, each one wrapped in antiseptic cotton. The hands and nails were thoroughly cleaned and white aprons donned by the surgeon and assistants. The instruments were placed in the usual porcelain and glass dishes and the necessary irrigators were in readiness. The operation was performed by Dr. Robert Abbé, by making a cross incision over the osteoma in the median line and dissecting back the muco-periosteum, exposing the hard surface of the tumor.

To save the shock to the brain, which might result from heavy chiselling, I used, at the request of Dr. Abbé, the dental engine and wheel-saw, cutting well into the base of the growth at several points.* Following this, forceps were applied to remove the bone, in pieces, with no shock whatever. The tumor had no pedicle and did not involve the vomer.

The muco-periosteum was then brought together, and an iodoform compress held in position by a silk ligature crossing from opposite twelfth-year molars. The tumor was of twenty years' growth, and from its peculiar position, I consider it of sufficient interest to present in this manner. We frequently find exostosis on both inferior and superior maxillary borders and exostosis of their cavities. They of course have a dental bearing, but this is the first instance I have met with of an eburne or ivory exostosis, involving only the median line and having no apparent connection with the alveolo-dental wall.

There was no special pathognomonic feature, as the patient had

Fig. 1.

Fig. 2.

not complained of any uneasiness in the tumor or of adjacent parts. The size of the tumor, however, had become an annoyance in eating and an impediment to speech. Its surface also had begun to ulcerate, painlessly. Dr. Abbé informs me that nine-tenths of the malignant growths on the tongue he has had to deal with are traceable to irritation due to ragged edges of teeth.

## Figures and Tables

**Fig. 1. f1:**
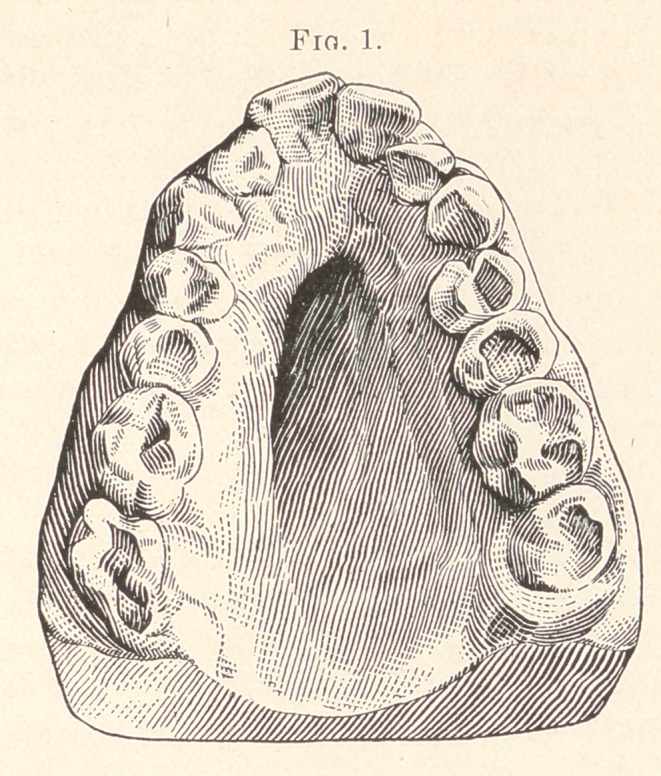


**Fig. 2. f2:**